# Quality of life in the people with disability: individual’s perception

**DOI:** 10.1192/j.eurpsy.2024.1227

**Published:** 2024-08-27

**Authors:** M. M. Matsumoto, D. Cardilli-Dias, D. R. Molini-Avejonas

**Affiliations:** Speech-Language Patology, University of São Paulo, São Paulo, Brazil

## Abstract

**Introduction:**

There are few studies about how people with intellectual disability (ID) perceive their own quality of life (QoL), with research being focus, mainly, in the opinion of caregivers and/or family. Thinking about QoL, the World Health Organization developed an instrument that measures QoL, the WHOQOL. In Brazil, this instrument was adapted, validated and translated for people with ID and their caregivers.

**Objectives:**

The aim of this study was to increase knowledge and understanding of how people with ID perceive their own QoL.

**Methods:**

This study was approved by the Ethics and Research Committee. Sample of 51 individuals aged between 19 and 54 years (G1), with medical diagnosis of ID, who did not present physical/mental disabilities and/or mental disorders and 31 caregivers (G2). G1 answered the WHOQOL-DIS-ID questionnaire and G2 answered the WHOQOL-DISID Proxy questionnaire. The results were statistically analyzed considering p-value ≤ 0.05.

**Results:**

The individuals with ID presented higher score on the psychological and lower score in the discrimination domain. The caregivers presented higher scores on the physical and lower scores in the autonomy domain. Regard the comparison between self-perception and the perception of caregivers, on Table 1, it was possible to observe significant differences in Psychological, Social, Environmental and all domains for the Disabilities Module.

**Table 1.** Correlation between Domains

Domain	Mean G1	Mean G2	SD G1	SD G2	Median G1	Median G2	P value
Physical Capacity	72,3	68,8	17,1	19,1	78,5	71,4	0,32
Psychological	80,1	67,7	17,5	17,9	83,3	66,6	<0,01*
Social Relationships	68,8	54,3	22,6	21,6	66,6	50	<0,01*
Environment	73,9	63,3	14,5	18,4	75	68,7	<0,01*
Discrimination	43,1	55,1	25,4	16,7	50	50	0,04*
Autonomy	65,5	41,9	31,4	25,2	66,6	50	<0,01*
Inclusion	77,0	58,9	16,1	20,1	75	54,1	<0,01*

**Conclusions:**

It is critical that people with ID participate in the creation and/or changes of inclusion policies and actions. Since the relationship between the perception of self-reported QoL and reported by caregivers are different and converge only in the physical domain.

**Disclosure of Interest:**

None Declared

